# Comparing timelines and evidence available to support new TB, HIV, and HCV drug approvals: The same, only different

**DOI:** 10.1371/journal.pone.0271102

**Published:** 2022-07-25

**Authors:** Allison LaHood, Rifat Rahman, Lindsay McKenna, Mike Frick, Carole D. Mitnick

**Affiliations:** 1 Department of Global Health and Social Medicine, Harvard Medical School, Boston, Massachusetts, United States of America; 2 Harvard Medical School, Boston, Massachusetts, United States of America; 3 Treatment Action Group, New York, New York, United States of America; HIV/STI Surveillance Research Center and WHO Collaborating Center for HIV Surveillance, Institute for Future Studies in Health, Kerman University of Medical Sciences, ISLAMIC REPUBLIC OF IRAN

## Abstract

**Background:**

Tuberculosis (TB), human immunodeficiency virus (HIV), and hepatitis C virus (HCV) share a global presence and propensity to disproportionately affect marginalized populations. However, over recent decades, many fewer drugs have been brought to market for TB than for the others. Although three new anti-TB drugs have been approved in the US or Europe in the last 10 years, uptake of these drugs has been limited. Using case examples of drugs developed recently for TB, HIV, and HCV, we explore possible reasons. We examine the use and effect of regulatory pathways intended to address weak economic incentives in the face of urgent, unmet needs; evaluate the extent of data underpinning authorizations for these indications; document development timelines and evidence available at the time of each approval; consider explanations for observed differences; and discuss the implications for clinical guidelines and use.

**Methods and findings:**

For each indication, we selected two drugs: one recently approved and one approved between 2012 and 2014, when the first new anti-TB drug from a novel class in more than 40 years received marketing authorization. We calculated time from first published peer-reviewed evidence of activity to date of approval; the number of phase 1, 2, and 3 trials; the number of trial participants randomized to treatment arms containing the drug; and the total number of participants in each trial from the individual drug approval packages. We found that the two TB drugs took longer to gain approval (8.0 and 19.2 years for bedaquiline and pretomanid, respectively) despite availing of special regulatory pathways meant to expedite approval, when compared to the HIV (2.6 years for dolutegravir and 4.7 years for doravirine) and HCV drugs (3.2 and 1.6 years for sofosbuvir and glecaprevir/pibrentasvir, respectively). Moreover, fewer participants were studied prior to TB drug approvals (380 and 879) than prior to approvals for HIV (1598 and 979) and for HCV (2291 and 2448) drugs.

**Conclusions:**

The dramatic disparities observed in TB drug development reaffirm the importance of several actions. Increased investment in TB research and development is necessary to rapidly advance drugs through the pipeline. Development plans and partnerships must provide safety and efficacy evidence on combinations and durations that are relevant to real-world use in heterogeneous populations. Reliable, validated surrogate markers of relapse-free cure are essential to decrease the duration and cost of TB treatment trials and increase the confidence and speed with which new regimens can advance. Lastly, regulators and normative bodies must maintain high evidentiary standards for authorization while ensuring timely and broad approval for TB drugs and regimens.

## Introduction

Tuberculosis (TB), human immunodeficiency virus (HIV), and hepatitis C virus (HCV) share some similarities, including their widespread distribution and propensity to disproportionately affect marginalized populations. The research and development landscapes for treatment of TB, HIV, and HCV, however, differ dramatically. Since 1987, when zidovudine (AZT) became the first treatment for HIV to gain the approval of the United States Food and Drug Administration (US FDA), 33 new chemical entities (NCEs) have been developed and approved for HIV [1]. These innovations have driven profound progress in the efficacy and safety of antiretroviral therapy (ART). The first decade following the approval of AZT and advent of highly active combination therapy saw the introduction of single-pill combination daily regimens. In the second decade, improved safety and efficacy of drug agents enabled the recommendation for universal ART (eliminating previous restrictions on treatment initiation based on CD4 cell count thresholds) [2]. The Joint United Nations Programme on HIV/AIDS credits improvements in treatment effectiveness and access to treatment for the important increase in the percentage of people living with HIV who experience viral suppression: up from 41% in 2015 to 59% in 2019 [3]. The access gap has also closed: of the 38 million people living with HIV in 2019, an estimated 25.4 million (67%) were receiving ART. This is more than triple the number of people receiving ART in 2010. Mortality has declined from over 1.2 million acquired immunodeficiency syndrome (AIDS)-related deaths in 2010 to 690,000 in 2019 [3].

With hepatitis C, change has been similarly pronounced on a more compressed timeline. Since 1989, when HCV was first isolated, 20 NCEs have been granted regulatory approval for the treatment of HCV. Before the 2011 introduction of boceprevir, the first direct-acting antiviral (DAA) approved for HCV, treatment was generally limited to long-term use of interferons, sometimes in combination with ribavirin. Outcomes were poor, with cure rates of only 50% [4]. With current HCV treatments, over 90% of people with chronic HCV can achieve cure in 2–3 months. Today, there is little to no new activity in the drug development pipeline of DAAs for HCV in light of the excellent profile of existing treatments: short duration, limited side effects, and 95% cure [5]. However, in contrast to HIV, the access gap is substantial; by the end of 2017, it was estimated that, of the 71 million people globally who had chronic HCV infection, only 5 million (7%) received treatment with DAAs [6]. Only nine countries are currently on track to reach World Health Organization (WHO) HCV elimination targets [7].

For TB, in contrast, over the last 40 years, only five NCEs (rifabutin, rifapentine, bedaquiline, delamanid, pretomanid) have been approved [8]. Improvements in the standard of care have been incremental. Treatment for drug-susceptible TB (DS-TB) still comprises four drugs administered for six months. Only recently, a phase 3 trial of a four-month regimen reported robust results establishing non-inferiority against the existing six-month standard of care [9]. Treatment for drug-resistant forms of TB (DR-TB) can last from 6 to 20 months; regimens comprise three to seven drugs, many of which have toxic side effects. Enormous access gaps remain: although TB is estimated to occur newly in more than 10 million people each year globally, less than 7 million are diagnosed and treated annually. Among patients newly affected by DR-TB that does not respond to first-line therapy, roughly 500,000 annually, only 35% receive appropriate diagnosis and treatment [10]. Tremendous variability is observed in TB treatment outcomes. While DS-TB is successfully treated in 85% globally; success is reported in only 76% of cases in Europe and the Americas. HIV coinfection also reduces probability of treatment success to below 60% in the Americas, Eastern Mediterranean, and Europe. Multidrug-resistant and rifampicin-resistant TB (MDR/RR-TB) treatment is successful in only 57% globally and as few as 52% of patients in Southeast Asia [10].

In view of the limited progress in the presence of overwhelming need for improvements in TB treatment, we interrogate the relative disparity between need and advances in therapeutics. Using case examples of drugs developed recently for TB, HIV, and HCV we explore possible reasons, including: 1) the use and effect of regulatory pathways intended to address weak economic incentives in the face of urgent unmet needs [11]; 2) the extent of data underpinning new United States Food and Drug Administration (FDA) and European Medicines Agency (EMA) drug approvals for these indications; 3) development timelines and extent of evidence available at the time of each approval. We consider explanations for observed differences and discuss the implications for clinical guidelines and use.

## Methods

For each of the three indications (TB, HIV, HCV), two drugs were systematically selected based on timing of approval by the FDA or EMA. We selected the NCEs that received approval most recently: pretomanid [12] for TB, glecaprevir/pibrentasvir [13] for HCV, and doravirine [14] for HIV. The second selection was timed to be contemporaneous with the FDA approval of bedaquiline [15] in 2012, the first new anti-TB drug from a novel class in more than 40 years. Contemporaneous selection was used to avoid bias resulting from different standards for drug approval in place at different times. For HIV, the NCE was dolutegravir [16] and for HCV, sofosbuvir [17]. For each drug, we collected the approval date; the number of phase 1, 2, and 3 trials; the number of trial participants randomized to treatment arms containing the NCE; the total number of participants in each trial; features of pivotal trials; postmarketing expectations; and regulatory pathways used for approval. Sources were the regulatory approval packages (medical and clinical review documents from Drugs@FDA database [18] and the European Public Assessment Report [EPAR] from the EMA marketing authorization database [19]). We confirmed phase 1 sample sizes in clinicaltrials.gov and, as necessary, we emailed the sponsor directly for participant numbers.

Time to first regulatory approval by the EMA or the FDA was calculated as the interval between the earliest peer-reviewed PubMed-indexed publication [20–25] reporting *in vitro* or clinical evidence of activity of the NCE against the relevant pathogen until the date of first approval by the US FDA or the EMA. Data were independently gathered and cross- checked by two reviewers (ANL, RR). The discrepancies noted were discussed with a third author (CDM) and resolved by consensus. Data were entered into MS Excel (v. 16.45). The figure was generated using RStudio, PBC (v. 1.3.1056, Vienna, Austria).

## Results

**Fig 1:**
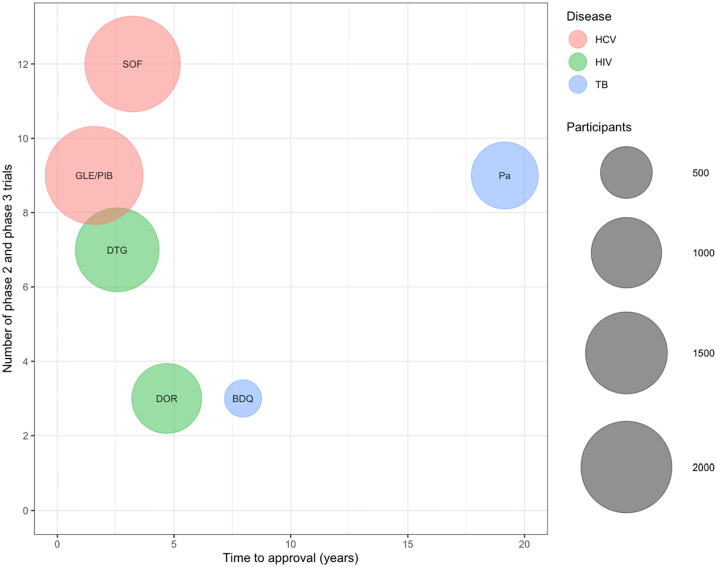
Number of participants, clinical trials, and time to first regulatory approval for each drug. Notes: The x-axis represents years from time to first published *in vitro* or clinical evidence of activity to approval. The y-axis illustrates the number of distinct phase 2 and phase 3 trials reported in the regulatory approval packages. [12–17] The bubbles are color coded by indication: blue for TB, green for HIV, and red for HCV. Their size (radius) is proportional to the total number of participants randomized (to receive the new chemical entity) across all phase 2 and phase 3 trials, as reported in the approval packages.

Selected drugs were bedaquiline (BDQ) and pretomanid (Pa, approved within a 3-drug regimen) for TB, dolutegravir (DTG) and doravirine (DOR) for HIV, and sofosbuvir (SOF) and glecaprevir/pibrentasvir (GLE/PIB) for HCV. Bedaquiline, dolutegravir, and sofosbuvir were first approved in 2012 or 2013. Pretomanid, doravirine, and glecaprevir/pibrentasvir were first approved for their respective indications between 2017 and 2019. Time from first peer-reviewed publication of demonstrated activity against the pathogen to first approval, number of phase 2/3 trials that informed authorization, and numbers of participants randomized to treatment arms containing the NCE before authorization are summarized in Fig 1 and detailed in S1 Table.

HCV and HIV drugs received regulatory approval faster than TB drugs. The range for HIV & HCV drugs was 1.6 to 4.7 years. Time to approval for bedaquiline was 8 years and for pretomanid almost 20 years. Approval packages for HCV drugs referenced the largest number of individual phase 2 and 3 clinical trials: 9 for sofosbuvir and 12 for glecaprevir/pibrentasvir. The HIV drug approval packages for doravirine and dolutegravir referenced 3 and 7 clinical trials, respectively. TB packages referenced 3 and 9 trials for bedaquiline and pretomanid, respectively. HCV drug (sofosbuvir and glecaprevir/pibrentasvir) trials referenced in the dossiers also included the most participants randomized to treatment arms containing the NCE: 2291 and 2448 participants randomized respectively. Referenced trials for HIV drugs (dolutegravir and doravirine) included 1598 and 979 participants. The TB drugs (bedaquiline and pretomanid) had the smallest number of participants randomized to treatment arms containing the NCE: 380 and 879 respectively across referenced trials. Similarly, fewer phase 1 trials with smaller numbers of participants were recorded for the TB drugs compared to HIV and HCV drugs (S2 Table).

The regulatory pathway to approval for each drug is described in Table 1. For the five drugs that were approved first by the FDA, three different pathways were used: accelerated approval, limited population for antibacterial and antifungal drugs (LPAD), and the traditional New Drug Application (NDA) approval. Bedaquiline qualified for accelerated FDA approval in combination with other effective (but unspecified) anti-TB drugs because the indication pursued, MDR-TB, was considered a serious condition and bedaquiline was deemed to fill an unmet medical need [26]. FDA accelerated approval of bedaquiline relied on a surrogate endpoint and required confirmatory phase 3 trials (Table 1). Pretomanid availed of the LPAD Pathway, which is reserved for serious or life-threatening infections in a limited population of patients with unmet needs. The approved indication is for the use of pretomanid only in combination with bedaquiline and linezolid in patients with extensively drug resistant (XDR) or treatment-nonresponsive or -intolerant MDR-TB [27]. The LPAD pathway permits subsequent applications, containing more evidence of safety and efficacy, to expand the indication or population. The third FDA pathway featured in Table 1, NDA approval (505(b)1 and 505(b)2), is the standard pathway; this mechanism requires substantial evidence of safety and efficacy before the FDA grants approval for U.S. commercialization. It was used for dolutegravir, doravirine, and sofosbuvir as single NCEs used in regimens with unspecified companion drugs. Glecaprevir/pibrentasvir was the only one of the NCEs examined here that was first approved by the EMA; its approval came through the EMA’s accelerated assessment pathway, which is distinct from the FDA’s accelerated approval. While the FDA “accelerates” by using a surrogate endpoint, the EMA expedites review: duration of evaluation is reduced from 210 to 150 days. EMA accelerated assessment is reserved for drugs that are of major public health interest [28].

**Table 1:** Regulatory pathways, study characteristics of pivotal clinical trials, and postmarketing expectations for selected TB, HIV, and HCV NCEs.

Drug	First FDA or EMA approval (year)	Regulatory pathway/licensing strategy	Pivotal trial characteristics	Indication on label	Postmarketing expectations^d^
bedaquiline [15]	FDA (2012)	Accelerated Approval Orphan Drug Designation	Phase 2 (NCT00449644) Randomized, placebo comparator Up to 96 weeks treatment Primary endpoints (surrogate) assessed at Weeks 8 and 24	Adult pulmonary MDR-TB, for use when an effective regimen cannot otherwise be provided	Conduct phase 3 RCT to assess relapse-free cure Establish patient registry to capture safety information Conduct in vitro studies to determine MIC Conduct in vitro study to determine effects on specific drug transporters Conduct DDI study with efavirenz Submit final report for phase 2 study, C208 Submit final report for phase 2 study, C209 *No pediatric study requirements (orphan drug designation).
pretomanid [12]	FDA (2019)	Limited Population Orphan Drug Designation	Phase 3 (NCT02333799) Single group assignment, no internal control 6–9 months treatment Primary endpoint (clinical) assessed 6 months after end of treatment	Adult pulmonary XDR-TB or TI/NR-MDR-TB, for use in limited and specific population, and only in combination with bedaquiline and linezolid	Conduct human semen study Establish global surveillance study to monitor for resistance Conduct PK and safety studies in people with renal impairment Conduct PK and safety studies in people with hepatic impairment Conduct rat carcinogenicity study Complete phase 3 ZeNix trial to optimize dose and duration of linezolid (when in combination with pretomanid and bedaquiline) Complete phase 3 SimpliciTB trial to determine safety and efficacy when used in combination with bedaquiline, moxifloxacin, and pyrazinamide to treat drug-sensitive TB *No pediatric study requirements (orphan drug designation).
dolutegravir [16]	FDA (2013)	NDA approval	Phase 3^a^ (NCT01231516) (NCT01227824) (NCT01263015) Randomized, active comparator 48–96 weeks treatment Primary endpoint (surrogate) assessed at Week 48	HIV-1 infection in adults and children aged 12 years and older and weighing at least 40 kg, for use in combination with other antiretroviral agents	Conduct a PK, safety, and antiviral activity trial in children 4 weeks to 12 years of age with HIV-1 infection that are integrase strand transfer inhibitor-naïve Conduct a PK, safety, and antiviral activity trial in children 2 years to 18 years of age with HIV-1 infection that are integrase strand transfer inhibitor (INSTI) experienced with certain INSTI associated resistance substitutions or that have clinically suspected INSTI resistance Submit final report from phase 3 trial in treatment experienced integrase strand transfer inhibitor-naïve PLHIV, ING111762 Submit final report from phase 3 trial in integrase strand transfer inhibitor-experienced PLHIV, ING112574 Submit final report from phase 3 trial in integrase strand transfer inhibitor-experienced PLHIV, ING116529 Conduct requested testing to evaluate drug substance and drug product impurities methods
doravirine [14]	FDA (2018)	NDA approval	Phase 3 (NCT02275780) (NCT02403674) Randomized, active comparator 96 weeks treatment Primary endpoint (surrogate) assessed at Week 48	HIV-1 infection in adult patients with no prior antiretroviral treatment history, for use in combination with other antiretroviral agents	Conduct PK, safety and antiviral activity studies in HIV-1 infected children: <18 years old and >35kg >2 years old and <35 kg 4 weeks to 23 months old Assess the phenotypic susceptibility in cell culture of doravirine and approved non-nucleoside reverse transcriptase inhibitors (NNRTIs) against Y318F alone and in combination with other substitutions Conduct a DDI study of doravirine and rifabutin
sofosbuvir [17]	FDA (2013)	NDA approval	Phase 3^b^ (NCT01497366) (NCT01542788) (NCT01604850) (NCT01682720) Randomized, active comparator OR placebo comparator 12–24 weeks treatment Primary endpoints (surrogate) assessed 12 weeks after end of treatment	Chronic hepatitis C infection in adults and children 12 years or older and weighing at least 35 kg, for use as a component of a combination of antiviral treatment regimen	Conduct PK, safety, and treatment response trial in children and adolescents 3–17 years old with chronic HCV Collect and analyze long-term safety data for children and adolescents enrolled in above study Submit final report for mouse carcinogenicity study Submit final report for rat carcinogenicity study Conduct short duration rat toxicology study Determine phenotypic susceptibility of sofosbuvir against various HCV genotypes with NS5B substitutions Submit final report and datasets including next generation sequencing for phase 2 trial, P7977-2025, to identify treatment-emergent substitutions and to obtain additional safety and efficacy data in people with hepatocellular carcinoma / awaiting liver transplantation Submit final report and datasets for phase 2b trial, GS-US-334-0154 (SOF + RBV), to provide dosing recommendations for HCV patients with impaired renal function and for HCV patients with ESRD Submit the final report and datasets for phase 3 trial, GS-US-334-0133 (SOF + RBV), in treatment naïve and experienced people with chronic genotype 2 or 3 HCV infection Submit the final report and datasets for phase 3 trial, GS-US-334-0123 (SOF + RBV), in people with chronic genotype 1, 2 and 3 HCV infection and HIV Submit the final report and datasets for phase 3 trial, GS-US-334-0109 (SOF + RBV +/- PEG), in people who participated in prior Gilead HCV studies Submit final report and datasets for phase 3b trial, GS-US-334-0153 (SOF + RBV +/- PEG), in people with genotype 2 or 3 chronic HCV infection Submit final report and datasets for phase 2 trial, GS-US-334-0126 (SOF + RBV), in people with recurrent chronic HCV post liver transplant Submit final report and datasets for phase 2 trial, GS-US-334-0125 (SOF + RBV), in people with cirrhosis and portal hypertension with or without liver decompensation Submit interim report from long-term observational study among people who achieve a sustained virologic response to treatment in Gilead-sponsored trials, GS-US-248-0122
glecaprevir/pibrentasvir [13]	EMA (2017)	Authorized after accelerated assessment	Phase 3^c^ (NCT02640157) (NCT02640482) Randomized, active comparator OR placebo comparator 8–12 weeks treatment Primary endpoints (surrogate) assessed 12 weeks after end of treatment	Chronic hepatitis C infection in adults and children aged 12 years or older	Submit periodic safety update reports Submit a Risk Management Plan (RMP) and perform the required pharmacovigilance activities and interventions detailed therein Conduct a non-interventional post-authorization safety study (PASS) to evaluate the recurrence of hepatocellular carcinoma associated with glecaprevir/pibrentasvir

^a^One phase 3 trial (NCT01328041) was a non-randomized, uncontrolled single group assignment.

^b^Other phase 3 trials were non-randomized, uncontrolled (NCT01667731) and single group assignment (NCT01641640).

^c^Other phase 3 trials were randomized, duration controlled (NCT02604017) and non-randomized, uncontrolled single group assignment (NCT02636595, NCT02642432, and NCT02651194).

^d^Includes both postmarketing commitments and postmarketing requirements for FDA approved drugs.

Abbreviations: TB = tuberculosis; HIV = human immunodeficiency virus; HCV = hepatitis C virus; NCE = new chemical entity; FDA = U.S. Food & Drug Administration; EMA = European Medicines Agency; MDR-TB = multidrug-resistant tuberculosis; RCT = randomized controlled trial; MIC = minimum inhibitory concentration; DDI = drug-drug interaction; XDR-TB extensively drug-resistant tuberculosis; TI/NR MDR-TB = treatment intolerant/non-responsive MDR-TB; PK = pharmacokinetic; PLHIV = people living with HIV; ESRD = end-stage renal disease; SOF = sofosbuvir; RBV = ribavirin; PEG = polyethylene glycol.

## Discussion

Here, we show that approvals for the two selected TB drugs took more time, despite having a foundation of fewer phase 2 and 3 clinical trials and participants, when compared to the HIV and HCV drugs. Although both TB NCEs availed of special regulatory pathways that are meant to expedite marketing authorization in case of important unmet health needs, 8 years elapsed for bedaquiline and 20 years for pretomanid between the first publication demonstrating anti-TB activity and the first marketing authorization by the US FDA or the EMA. This suggests that other barriers remain throughout the TB NCE development cycle which delay time to registration.

Some reasons that have previously been used to explain the long timelines observed for the development of TB treatments [29, 30] are not unique to TB. First, the high efficacy of the standard-of-care TB treatment is often cited as a cause for the lengthy development period. Demonstrating superiority over a highly effective treatment requires a very large sample size, which, in turn, requires extended recruitment periods and/or large numbers of trial sites. This also applies to HIV and HCV. Notably, in HCV, the current standard of care is estimated to cure 95% of patients [31]. In trials of drug-susceptible TB, generally, more than 85% of participants in the control arm experience successful outcomes [32–34]. Because of the high efficacy, these require large sample sizes. To avoid the time and cost consequences of such large sample sizes, developers of bedaquiline and pretomanid ultimately chose DR-TB as the indication for pivotal trials supporting approval of these NCEs. With historically poor efficacy [10], it was considerably easier to demonstrate an improvement over the existing standard of care for DR-TB than for DS-TB. Although this choice was expected to shorten the time to marketing authorization, the interval for these anti-TB drugs still exceeded those for HIV and HCV NCEs developed in the same era.

Second, enrollment period aside, trials of TB treatment are often noted to be long [29, 35, 36]. In part this is due to the protracted treatment required to eliminate the notoriously slow-growing *M*. *tuberculosis*: historically 6 months for drug-susceptible (DS) TB and 18–24 months for drug-resistant (DR) TB [37, 38]. In addition, extended follow-up is necessary to detect relapse. In accordance with FDA and EMA guidance, at least two years of post-randomization follow-up were required in the pivotal trials of bedaquiline and pretomanid [39, 40]. Final outcome assessment was similarly protracted in the pivotal trials of dolutegravir and doravirine. Only the pivotal trials of sofosbuvir and glecaprevir/pibrentasvir had shorter follow-up periods, 9 months or less for all efficacy endpoints. This may contribute to the longer timeline for TB compared to HCV drug approvals; it does not, however, explain the difference between TB and HIV.

A third, related reason that has previously been offered is the absence of a validated surrogate marker [41] for clinical endpoints in TB while viral load fulfills this function for both HIV and HCV [42]. As noted above, this difference did not materially affect the relative expediency of assessing final efficacy endpoints in pivotal HIV and TB trials. It may, however, differentially affect earlier-stage clinical testing and the interval between start of phase 2 and phase 3 testing. A reliable marker, similar to HIV viral load, used to demonstrate efficacy against TB in phase 2 trials, can lead to earlier and more confident initiation of phase 3 trials. Notably, in three recent phase 3 trials of new TB treatments, early treatment response in phase 2 trials did not predict overall performance of shortened regimens [32–34].

Among these three reasons previously advanced to explain the protracted nature of TB drug development, only the absence of a validated surrogate endpoint appears to be a likely, important contributor to the overall longer development timeline for TB. The extended interval to final outcome assessment was common among the three indications. In contrast, the choice of drug-resistant TB as the indication logically should accelerate anti-TB NCE development, because of the smaller required sample size to establish superiority or non-inferiority. Although this choice did not result in timelines comparable to those for HIV and HCV, it likely contributed to the smaller numbers of subjects involved in overall clinical development of the anti-TB drugs. Other possible explanations exist for the combination of relatively longer timeline and less-complete evidence characterizing TB drug development efforts. Below we explore these and their consequences on uptake of the new drugs.

Pretomanid and bedaquiline both availed of regulatory measures that likely reduced their time to approval. Both received orphan designation according to the Orphan Drug Act, which induces development of treatments for conditions that affect fewer than 200,000 people in the US. Both also availed of alternative FDA pathways, LPAD for pretomanid and Accelerated Approval for bedaquiline. In exchange for addressing an urgent unmet medical need, these pathways relax the pre-approval requirements for completeness and rigor of evidence compared to standard pathways for marketing authorization. Bedaquiline, for example, received accelerated approval based only on phase 2b data with the expectation that a phase 3 trial be completed post-approval; the resulting phase 3 trial is expected to report in 2022, 10 years after initial approval [43]. The LPAD pathway approval of pretomanid as part of three-drug regimen relied on efficacy data from just 109 patients enrolled in a single-arm study. There was no internal, concurrent control [44]. This is distinct from the standard pathway for approval, which normally requires at least one phase 3 randomized trial(s) with an internal, concurrent control. This deviation from the gold standard is, however, common for alternative pathways. A review of FDA approvals granted for novel therapeutics between 2005 and 2012 found that accelerated approvals and approvals of orphan drugs had fewer pivotal trials and participants than drugs approved through the standard pathway. And, the pivotal trials supporting accelerated approvals and approvals of orphan drugs were less likely to have randomized, controlled, double-blinded designs [45]. Sponsors may struggle to justify investments in expensive, high quality RCTs that take longer to complete, especially if there is a “faster” way to market.

Another complicating factor for—but not unique to—TB is its requirement for multidrug treatment. The efficacy and safety of new drugs must be tested in combination with other drugs. If these are other NCEs, regimen development requires that all products in the combination are developed by the same entity or that multiple developers are willing to collaborate. This entails deviation from the typical approach to drug development, which is generally guided by protection of intellectual property. Initiatives such as the Project to Accelerate New Treatments for Tuberculosis (PAN-TB), the European Regimen Accelerator for Tuberculosis (ERA4TB), and Academia and Industry United Innovation and Treatment for Tuberculosis (UNITE4TB) have been established to facilitate collaboration and to shorten the timeline to TB regimen development.

Optimizing composition, dosing, and duration of drug combinations for safety and efficacy is decidedly complex and has important implications for uptake [30, 46]. Typically, single drugs are studied in phase 1 and early phase 2 trials. Limited combinations are studied first in animal models. Selection and duration of combinations are translated from animal to phase 2 and phase 3 trials of efficacy and safety. Gaps in knowledge often persist about the contribution of individual drugs to a multidrug regimen and about variable effectiveness of regimen durations in a heterogeneous population. Against this backdrop, several large phase 3 trials have failed to demonstrate non-inferiority of tested regimens, despite encouraging results in animal and phase 2 studies [32–34, 47]. To avoid these outcomes, novel approaches to, and an increased focus on, preclinical research, translational models, and phase 2 TB trials relying on yet-to-be validated markers have been proposed [48–50]. This approach is intended to accelerate development of TB regimens comprising multiple NCEs (especially if they are first in class or have a novel mechanism of action), generate complete information on the individual components, and increase probability of success of late-stage clinical studies.

Moreover, this comprehensive approach can facilitate uptake of new anti-TB drugs following regulatory approval if it generates real-world evidence on the use of NCEs in regimens, at durations, and in patients that reflect the priorities of health-care systems and the populations they serve. Also key to adoption is sufficient numbers of individuals exposed to novel drugs and regimens to provide confidence that important rare events have not gone undetected or misestimated. Bedaquiline, which received marketing authorization based on the smallest number of study participants and trials among the NCEs examined here, has faced constraints to its use [46, 51]. It is clear from this experience, together with that on the aforementioned limited compliance with postmarketing expectations for orphan and accelerated approvals [52], that the pre-approval window is the optimal time to provide complete research on the individual components, their combination, and their use in target populations [46, 52].

The limited evidence for new anti-TB treatments also undermines uptake in another way. TB practice is highly influenced by WHO guidance, which emerges from reviews of available evidence. Examined by the WHO Guidelines Development Group, the evidence that led to FDA approvals of bedaquiline and the pretomanid-containing regimen was graded as “very low certainty.” Recommendations for their use were conditional [53, 54]. Historically, weak evidence that produces conditional recommendations has been associated with delayed policy and practice changes by national TB programs, even for the broader populations [55]. Uptake delays for special populations, i.e., children, pregnant people, those with comorbidities are considerably more delayed by the absence of evidence on these populations. Ultimately, the regulatory pathways intended to incentivize and accelerate the development of new drugs to address an unmet need have failed to close the gap in the development timeline. Moreover, they have inadvertently weakened the body of evidence that is necessary for policymakers and healthcare providers to introduce the new products with confidence. This exacerbates delays in access for patients in need.

The findings of the present study are likely linked to the fact that funding for TB research consistently falls short of need. The gap between estimated funding required to achieve the sustainable development goals for TB and actual funding is $1.1 billion [56]. Two-thirds of funding for TB research comes from the public sector [56]. Even in the realm of philanthropic and public-sector investment, TB receives less research funding than HIV (and commensurate funding to HCV) relative to their respective burdens, as measured by 2017 research dollar per disability-adjusted life-year (DALY) and death, $156/DALY for TB, $155/DALY for HCV, and $772/DALY for HIV [57].

Despite the aforementioned incentives, and their significant financial value to developers [58], industry contribution to TB R&D is paltry, estimated to be less than 10% of total expenditures in recent years, and declining [59]. Advocates, scientists, and policymakers have called for innovative financing models to address the challenge of limited industry investment in TB drug development and antibiotic research more broadly [60]. One such proposed model, the Life Prize, sought to develop new regimens for TB by deploying a mixture of push and pull incentives to foster collaboration among different developers, who would agree to pool compounds, data, and intellectual property in exchange for push funding and pull prizes [51]. The idea was that by delinking the costs of R&D from the sales volumes and prices of final products, new TB drugs could be developed together as regimens under a framework that would reward the collaboration required to generate data on individual drugs within multi-drug combinations, answer the research questions meaningful to TB patients and programs, and satisfy regulatory requirements for licensure. Since governments and other donors did not step up to support the Life Prize, the need remains for financial models that can accelerate TB research in ways that generate high quality evidence for regulatory approval, normative guidance, and public health practice.

Our study has some potential limitations. It is possible that the HIV and HCV drugs chosen were outliers, approved more quickly and/or with more patients than other NCEs for these indications. To minimize the risk of bias due to secular changes in drug approval standards over time, we selected drugs approved in the same time periods as bedaquiline and pretomanid. Our choice of the date of the first peer-reviewed publication reporting activity against the pathogen as the starting point to assess time to regulatory approval could introduce bias. If, for example, HIV or HCV (but not TB) drugs had demonstrated activity long before the first publication, then the interval to TB drug approval, relative to that of the other drugs, could be overestimated. Since the interval for TB drugs was so much longer than for HIV and HCV (3.3 years minimum between doravirine and bedaquiline), a relative publication delay of even 2 years for HIV or HCV results would not erase the difference.

## Conclusion

Existing incentives offered under the Orphan Drug Act and special regulatory pathways for conditions of unmet medical need have been insufficient to overcome the long delays between demonstration of anti-TB activity and licensure. Moreover, when used to obtain approval of TB drugs, these incentives resulted in reduced quantity and rigor of clinical evidence. With 15 compounds in clinical development, including 8 that pursue 5 novel targets, the TB treatment pipeline is fuller than it has been in decades. The great opportunities presented by the TB treatment pipeline juxtaposed against the dramatic disparities described in this study reaffirm the importance of several actions. First, the situation demands increased private and public investments in TB R&D, as well as improved collaboration among developers to produce evidence that: 1) expedites the pace at which new drugs and regimens advance through the pipeline; 2) ensures the availability of complete information on individual components and full regimens; and 3) facilitates uptake of new regimens in populations that require them. This includes collaboration among commercial developers and non-commercial agents when necessary to complete the portfolio of real-world evidence [46]. Second, innovation in research tools, including validated, reliable surrogate markers of relapse-free cure, are critical to decrease both the duration and cost of future TB treatment trials and to increase the confidence and speed with which new regimens can advance through stages of clinical research. Finally, regulators and normative bodies must maintain high evidentiary standards for new TB drugs and regimens and their timely approval for broad use. Only with these advances can we quicken the pace of TB drug development while also ensuring that people affected by TB receive treatment backed by the highest standard of safety and efficacy data. The needs of the 10 million people who fall sick with TB each year must guide the optimal approach to the resources in the TB pipeline.

## Supporting information

S1 Table Number of participants, clinical trials and time to first regulatory approval for each drug. (DOCX)

S2 Table Phase 1 trials referenced in earliest approved FDA or EMA approval packages for each drug. (DOCX)

## References

[1] FDA-approved HIV medicines [Internet]. HIVinfo.NIH.gov. 2021 [cited 2021 Dec 14]. https://hivinfo.nih.gov/understanding-hiv/fact-sheets/fda-approved-hiv-medicines

[2] Horn T, Collins S. The antiretroviral pipeline [Internet]. New York, New York: Treatment Action Group; 2016 Jul. https://www.treatmentactiongroup.org/resources/pipeline-report/2016-pipeline-report/the-antiretroviral-pipeline-3/

[3] 2020 Global AIDS Update: Seizing the moment—Tackling entrenched inequalities to end epidemics [Internet]. Geneva, Switzerland: UNAIDS; 2020. https://www.unaids.org/en/resources/documents/2020/global-aids-report

[4] WHO Global report on access to hepatitis C treatment—Focus on overcoming barriers [Internet]. Geneva, Switzerland: World Health Organization; 2016. Report No.: WHO/HIV/2016.20. http://www.who.int/hepatitis/publications/hep-c-access-report/en/

[5] Gaudino A. HCV Treatment Pipeline 2020 [Internet]. New York, New York: Treatment Action Group; 2020. https://www.treatmentactiongroup.org/resources/pipeline-report/2020-pipeline-report/

[6] Accelerating access to hepatitis C diagnostics and treatment: overcoming barriers in low and middle-income countries. Global progress report 2020 [Internet]. Geneva, Switzerland: World Health Organization; 2020. https://www.who.int/publications/i/item/9789240019003

[7] Polaris Observatory [Internet]. CDA Foundation. [cited 2021 Mar 31]. https://cdafound.org/polaris/

[8] Global investments in tuberculosis research and development: Past, present, and future [Internet]. Geneva: World Health Organization; 2017. 80 p. http://www.who.int/tb/publications/2017/Global_Investments_in_Tuberculosis_Research_Investment/en/

[9] Dorman SE, Nahid P, Kurbatova EV, Phillips PPJ, Bryant K, Dooley KE, et al. Four-month rifapentine regimens with or without moxifloxacin for tuberculosis. N Engl J Med. 2021 May 6;384(18):1705–18. doi: 10.1056/NEJMoa2033400 33951360 PMC8282329

[10] Global tuberculosis report 2020 [Internet]. Geneva: World Health Organization; 2020. 232 p. https://www.who.int/publications-detail-redirect/9789240013131

[11] Trouiller P, Olliaro P, Torreele E, Orbinski J, Laing R, Ford N. Drug development for neglected diseases: a deficient market and a public-health policy failure. Lancet. 2002 Jun 22;359(9324):2188–94. doi: 10.1016/S0140-6736(02)09096-7 12090998

[12] Drug Approval Package: Pretomanid [Internet]. U.S. Food & Drug Administration. 2019. https://www.accessdata.fda.gov/drugsatfda_docs/nda/2019/212862Orig1s000TOC.cfm

[13] Maviret (glecaprevir/pibrentasvir): EMA authorisation details [Internet]. European Medicines Agency. 2018. https://www.ema.europa.eu/en/medicines/human/EPAR/maviret

[14] Drug Approval Package: PIFELTRO (doravirine) [Internet]. U.S. Food & Drug Administration. 2018. https://www.accessdata.fda.gov/drugsatfda_docs/nda/2018/210806Orig1s000,210807Orig1s000TOC.cfm

[15] Drug Approval Package: SIRTURO (bedaquiline) [Internet]. U.S. Food & Drug Administration. 2013. https://www.accessdata.fda.gov/drugsatfda_docs/nda/2012/204384Orig1s000TOC.cfm

[16] Drug Approval Package: TIVICAY (dolutegravir) [Internet]. U.S. Food & Drug Administration. 2013. https://www.accessdata.fda.gov/drugsatfda_docs/nda/2013/204790Orig1s000TOC.cfm

[17] Drug Approval Package: SOVALDI (sofosbuvir) [Internet]. U.S. Food & Drug Administration. 2014. https://www.accessdata.fda.gov/drugsatfda_docs/nda/2013/204671orig1s000toc.cfm

[18] Drugs@FDA: FDA-Approved Drugs [Internet]. U.S. Food & Drug Administration. 2021 [cited 2021 Apr 5]. https://www.accessdata.fda.gov/scripts/cder/daf/

[19] Medicines [Internet]. European Medicines Agency. [cited 2021 Apr 5]. https://www.ema.europa.eu/en/medicines

[20] Andries K, Verhasselt P, Guillemont J, Göhlmann HWH, Neefs JM, Winkler H, et al. A diarylquinoline drug active on the ATP synthase of Mycobacterium tuberculosis. Science. 2005 Jan 14;307(5707):223–7. doi: 10.1126/science.1106753 15591164

[21] Stover CK, Warrener P, VanDevanter DR, Sherman DR, Arain TM, Langhorne MH, et al. A small-molecule nitroimidazopyran drug candidate for the treatment of tuberculosis. Nature. 2000 Jun 22;405(6789):962–6. doi: 10.1038/35016103 10879539

[22] Sofia MJ, Bao D, Chang W, Du J, Nagarathnam D, Rachakonda S, et al. Discovery of a β-d-2’-deoxy-2’-α-fluoro-2’-β-C-methyluridine nucleotide prodrug (PSI-7977) for the treatment of hepatitis C virus. J Med Chem. 2010 Oct 14;53(19):7202–18. doi: 10.1021/jm100863x 20845908

[23] Côté B, Burch JD, Asante-Appiah E, Bayly C, Bédard L, Blouin M, et al. Discovery of MK-1439, an orally bioavailable non-nucleoside reverse transcriptase inhibitor potent against a wide range of resistant mutant HIV viruses. Bioorg Med Chem Lett. 2014 Feb 1;24(3):917–22. doi: 10.1016/j.bmcl.2013.12.070 24412110

[24] Kobayashi M, Yoshinaga T, Seki T, Wakasa-Morimoto C, Brown KW, Ferris R, et al. In Vitro antiretroviral properties of S/GSK1349572, a next-generation HIV integrase inhibitor. Antimicrob Agents Chemother. 2011 Feb;55(2):813–21. doi: 10.1128/AAC.01209-10 21115794 PMC3028777

[25] Lawitz EJ, O’Riordan WD, Asatryan A, Freilich BL, Box TD, Overcash JS, et al. Potent antiviral activities of the direct-acting antivirals ABT-493 and ABT-530 with three-day monotherapy for Hepatitis c virus genotype 1 infection. Antimicrob Agents Chemother. 2015 Dec 28;60(3):1546–55. doi: 10.1128/AAC.02264-15 26711747 PMC4775945

[26] Accelerated Approval Program [Internet]. U.S. Food & Drug Administration. FDA; 2020. https://www.fda.gov/drugs/information-health-care-professionals-drugs/accelerated-approval-program

[27] Limited population pathway for antibacterial and antifungal drugs–The LPAD pathway [Internet]. U.S. Food & Drug Administration. FDA; 2020. https://www.fda.gov/drugs/development-resources/limited-population-pathway-antibacterial-and-antifungal-drugs-lpad-pathway

[28] Accelerated assessment [Internet]. European Medicines Agency. European Medicines Agency; 2021. https://www.ema.europa.eu/en/human-regulatory/marketing-authorisation/accelerated-assessment

[29] Phillips PPJ, Mitnick CD, Neaton JD, Nahid P, Lienhardt C, Nunn AJ. Keeping phase III tuberculosis trials relevant: Adapting to a rapidly changing landscape. PLoS Med. 2019 Mar;16(3):e1002767. doi: 10.1371/journal.pmed.1002767 30901331 PMC6430373

[30] Brigden G, Nyang’wa BT, du Cros P, Varaine F, Hughes J, Rich M, et al. Principles for designing future regimens for multidrug-resistant tuberculosis. Bull World Health Organ. 2014 Jan 1;92(1):68–74. doi: 10.2471/BLT.13.122028 24391302 PMC3865549

[31] Fact sheet: Hepatitis C [Internet]. World Health Organization. 2020 [cited 2021 May 17]. https://www.who.int/news-room/fact-sheets/detail/hepatitis-c

[32] Jindani A, Harrison TS, Nunn AJ, Phillips PPJ, Churchyard GJ, Charalambous S, et al. High-dose rifapentine with moxifloxacin for pulmonary tuberculosis. N Engl J Med. 2014 Oct 23;371(17):1599–608. doi: 10.1056/NEJMoa1314210 25337749 PMC4233406

[33] Gillespie SH, Crook AM, McHugh TD, Mendel CM, Meredith SK, Murray SR, et al. Four-month moxifloxacin-based regimens for drug-sensitive tuberculosis. N Engl J Med. 2014 Oct 23;371(17):1577–87. doi: 10.1056/NEJMoa1407426 25196020 PMC4277680

[34] Merle CS, Fielding K, Sow OB, Gninafon M, Lo MB, Mthiyane T, et al. A four-month gatifloxacin-containing regimen for treating tuberculosis. N Engl J Med. 2014 Oct 23;371(17):1588–98. doi: 10.1056/NEJMoa1315817 25337748

[35] Lienhardt C, Raviglione M, Spigelman M, Hafner R, Jaramillo E, Hoelscher M, et al. New drugs for the treatment of tuberculosis: needs, challenges, promise, and prospects for the future. The Journal of Infectious Diseases. 2012 May 15;205 Suppl 2:S241–249. 22448022 10.1093/infdis/jis034

[36] Ginsberg AM, Spigelman M. Challenges in tuberculosis drug research and development. Nature Medicine. 2007 Mar;13(3):290–4. doi: 10.1038/nm0307-290 17342142

[37] Guidelines for treatment of drug-susceptible tuberculosis and patient care, 2017 update [Internet]. Geneva: World Health Organization; 2017. 56 p. https://apps.who.int/iris/bitstream/handle/10665/255052/9789241550000-eng.pdf

[38] WHO treatment guidelines for drug-resistant tuberculosis, 2016 update [Internet]. Geneva: World Health Organization; 2016 [cited 2021 May 21]. https://www.who.int/publications-detail-redirect/9789241549639 27748093

[39] Pulmonary tuberculosis: Developing drugs for treatment [Internet]. FDA Center for Drug Evaluation and Research; 2013 Nov. Report No.: FDA-2013-D-1319. https://www.fda.gov/regulatory-information/search-fda-guidance-documents/pulmonary-tuberculosis-developing-drugs-treatment

[40] Addendum to the guideline on the evaluation of medicinal products indicated for treatment of bacterial infections to address the clinical development of new agents to treat pulmonary disease due to Mycobacterium tuberculosis [Internet]. European Medicines Agency; 2017 Jul. Report No.: EMA/CHMP/EWP/14377/2008 Rev. 1. https://www.ema.europa.eu/en/addendum-note-guidance-evaluation-medicinal-products-indicated-treatment-bacterial-infections

[41] Surrogate endpoint resources for drug and biologic development [Internet]. U.S. Food & Drug Administration. FDA; 2018 [cited 2021 Mar 4]. https://www.fda.gov/drugs/development-resources/surrogate-endpoint-resources-drug-and-biologic-development

[42] Table of surrogate endpoints that were the basis of drug approval or licensure [Internet]. U.S. Food & Drug Administration. FDA; 2021 [cited 2021 Apr 5]. https://www.fda.gov/drugs/development-resources/table-surrogate-endpoints-were-basis-drug-approval-or-licensure

[43] Diacon AH, Pym A, Grobusch MP, de los Rios JM, Gotuzzo E, Vasilyeva I, et al. Multidrug-resistant tuberculosis and culture conversion with bedaquiline. N Engl J Med. 2014 Aug 21;371(8):723–32. doi: 10.1056/NEJMoa1313865 25140958

[44] Conradie F, Diacon AH, Ngubane N, Howell P, Everitt D, Crook AM, et al. Treatment of Highly Drug-Resistant Pulmonary Tuberculosis. N Engl J Med. 2020 Mar 5;382(10):893–902. doi: 10.1056/NEJMoa1901814 32130813 PMC6955640

[45] Downing NS, Aminawung JA, Shah ND, Krumholz HM, Ross JS. Clinical trial evidence supporting FDA approval of novel therapeutic agents, 2005–2012. JAMA. 2014 Jan 22;311(4):368–77. doi: 10.1001/jama.2013.282034 24449315 PMC4144867

[46] Perrin C, Athersuch K, Elder G, Martin M, Alsalhani A. Recently developed drugs for the treatment of drug-resistant tuberculosis: a research and development case study. BMJ Glob Health. 2022 Apr;7(4):e007490. doi: 10.1136/bmjgh-2021-007490 35440441 PMC9020285

[47] Guglielmetti L, Low M, McKenna L. Challenges in TB regimen development: preserving evidentiary standards for regulatory decisions and policymaking. Expert Rev Anti Infect Ther. 2020 Aug;18(8):701–4. doi: 10.1080/14787210.2020.1756776 32345064

[48] Davies G, Boeree M, Hermann D, Hoelscher M. Accelerating the transition of new tuberculosis drug combinations from Phase II to Phase III trials: New technologies and innovative designs. PLoS Med. 2019 Jul;16(7):e1002851. doi: 10.1371/journal.pmed.1002851 31287813 PMC6615592

[49] Lienhardt C, Nunn A, Chaisson R, Vernon AA, Zignol M, Nahid P, et al. Advances in clinical trial design: Weaving tomorrow’s TB treatments. PLoS Med. 2020 Feb;17(2):e1003059. doi: 10.1371/journal.pmed.1003059 32106220 PMC7046183

[50] Ernest JP, Strydom N, Wang Q, Zhang N, Nuermberger E, Dartois V, et al. Development of New Tuberculosis Drugs: Translation to Regimen Composition for Drug-Sensitive and Multidrug-Resistant Tuberculosis. Annu Rev Pharmacol Toxicol. 2021 Jan 6;61:495–516. doi: 10.1146/annurev-pharmtox-030920-011143 32806997 PMC7790895

[51] Brigden G, Castro JL, Ditiu L, Gray G, Hanna D, Low M, et al. Tuberculosis and antimicrobial resistance—new models of research and development needed. Bull World Health Organ. 2017 May 1;95(5):315. doi: 10.2471/BLT.17.194837 28479629 PMC5418818

[52] Moneer O, Brown BL, Avorn J, Darrow JJ, Mitra-Majumdar M, Joyce KW, et al. New drug postmarketing requirements and commitments in the US: a systematic review of the evidence. Drug Saf. 2022 Apr;45(4):305–18. doi: 10.1007/s40264-022-01152-9 35182362

[53] WHO consolidated guidelines on tuberculosis Module 4: Treatment—Drug-resistant tuberculosis treatment [Internet]. Geneva: World Health Organization; 2020. https://www.who.int/publications-detail-redirect/9789240007048 32603040

[54] The use of bedaquiline in the treatment of multidrug-resistant tuberculosis: Interim policy guidance [Internet]. Geneva: World Health Organization; 2013. http://www.ncbi.nlm.nih.gov/books/NBK154134/23967502

[55] Nasser SMU, Cooke G, Kranzer K, Norris SL, Olliaro P, Ford N. Strength of recommendations in WHO guidelines using GRADE was associated with uptake in national policy. J Clin Epidemiol. 2015 Jun;68(6):703–7. doi: 10.1016/j.jclinepi.2014.11.006 25578218

[56] Tomlinson C. Tuberculosis research funding trends, 2005–2020 [Internet]. New York, New York: Treatment Action Group; 2021. https://www.treatmentactiongroup.org/wp-content/uploads/2021/12/tb_funding_2021.pdf

[57] Head MG, Brown RJ, Newell ML, Scott JAG, Batchelor J, Atun R. The allocation of USdollar;105 billion in global funding from G20 countries for infectious disease research between 2000 and 2017: a content analysis of investments. Lancet Glob Health. 2020 Oct;8(10):e1295–304. doi: 10.1016/S2214-109X(20)30357-0 32971052 PMC7505652

[58] Ridley DB, Régnier SA. The commercial market for priority review vouchers. Health Aff (Millwood). 2016 May 1;35(5):776–83. doi: 10.1377/hlthaff.2015.1314 27140982

[59] Gotham D, McKenna L, Frick M, Lessem E. Public investments in the clinical development of bedaquiline. PLoS One. 2020;15(9):e0239118. doi: 10.1371/journal.pone.0239118 32946474 PMC7500616

[60] Lienhardt C, Zumla A, Gebreselassie N, Frick M, Gray G, Kasaeva T, et al. Tuberculosis research and development: seeding the future. Lancet Respir Med. 2018 Apr;6(4):242–4. doi: 10.1016/S2213-2600(18)30050-X 29595503

