# Supplementary material for: Comparing timelines and evidence available to support new TB, HIV, and HCV drug approvals: The same, only different
Source: PLoS One. 2022 Jul 25;17(7):e0271102. doi: 10.1371/journal.pone.0271102 (PMC9312388; doi:10.1371/journal.pone.0271102)
Supplement: S1 Table — (DOCX) [file pone.0271102.s001.docx]

| Drug | Disease | Total Participants | Participants randomized to treatment arms containing NCE | # Phase 2/3 trials | Date identified as treatment^a^ | Date of first regulatory approval | Time to approval (years)^b^ |
| --- | --- | --- | --- | --- | --- | --- | --- |
| bedaquiline | TB | 515 | 380 | 3 | 2005-01-14 | 2012-12-28 | 8.0 |
| pretomanid | TB | 1183 | 879 | 9 | 2000-06-22 | 2019-08-14 | 19.2 |
| dolutegravir | HIV | 2848 | 1598 | 7 | 2011-01-20 | 2013-08-13 | 2.6 |
| doravirine | HIV | 1834 | 979 | 3 | 2013-12-23 | 2018-08-30 | 4.7 |
| sofosbuvir | HCV | 2752 | 2291 | 12 | 2010-09-16 | 2013-12-06 | 3.2 |
| glecaprevir/pibrentasvir | HCV | 2663 | 2448 | 9 | 2015-12-28 | 2017-07-26 | 1.6 |

**S1 Table. Number of participants, clinical trials and time to first regulatory approval for each drug.**

^a^Date of the earliest peer-reviewed publication with evidence of in vitro or clinical drug activity against the relevant pathogen

^b^Years between the date identified as treatment and the date of first regulatory approval

Note: NCE = new chemical entity; TB = tuberculosis; HIV = human immunodeficiency virus; HCV = hepatitis C virus
