# Supplementary material for: Comparing timelines and evidence available to support new TB, HIV, and HCV drug approvals: The same, only different
Source: PLoS One. 2022 Jul 25;17(7):e0271102. doi: 10.1371/journal.pone.0271102 (PMC9312388; doi:10.1371/journal.pone.0271102)
Supplement: S2 Table — (DOCX) [file pone.0271102.s002.docx]

**S2 Table. Phase 1 trials referenced in earliest approved FDA or EMA approval packages for each drug**

| Drug | # of phase 1 Trials | # of phase 1 participants (including placebo/control) | # of phase 1 participants dosed with each drug |
| --- | --- | --- | --- |
| bedaquiline | 11 | 337 | 265 |
| pretomanid | 10 | 324 | 289 |
| dolutegravir | 22 | 390 | 376 |
| doravirine | 20 | 704 | 678 |
| sofosbuvir | 13 | 484 | 451 |
| glecaprevir/pibrentasvir | 33 | 1047 | 1027 |
